# Quantitative Analysis of the Efficacy of PARP Inhibitors as Maintenance Therapy in Recurrent Ovarian Cancer

**DOI:** 10.3389/fphar.2021.771836

**Published:** 2021-11-08

**Authors:** Lili Gao, Rui Chen, Ting Li, Lujin Li, Qingshan Zheng

**Affiliations:** Center for Drug Clinical Research, Shanghai University of Traditional Chinese Medicine, Shanghai, China

**Keywords:** recurrent ovarian cancer, maintenance treatment, PARP, pharmacodynamic model, progress-free survival

## Abstract

**Objective:** This study aimed to establish a pharmacodynamic model and to screen reasonable covariates to quantitatively describe the efficacy of poly (ADP-ribose) polymerase inhibitors (PARPis) as maintenance treatment for recurrent ovarian cancer (ROC).

**Methods:** The log normal hazard function model was established by using progression-free survival (PFS) data of 1,169 patients from published randomized trials on FDA-approved PARP inhibitors (olaparib, niraparib, and rucaparib). Monte Carlo simulation was used to compare PFS values in different scenarios, such as monotherapy (administered alone) and combination therapy (PARPis combined with chemo- or target-therapies), different biomarker statuses, and different PARP inhibitors. PFS was also estimated.

**Results:** The study showed that the median PFS was 8.5 months with monotherapy and 16.0 months with combination therapy. The median PFS of patients with the BRCA mutation, BRCA wild-type, and HRD-positivity were 11.0, 7.5, and 9.0 months in monotherapy, respectively, and 23.0, 14.0 and 17.5 months, in combination therapy, respectively. In addition, the median PFS of olaparib, niraparib, and rucaparib monotherapy were about 9.5, 10.5, and 12.0 months, respectively, and about 19.0, 20.0, and 25 months, respectively, in combination therapy. The median PFS values in combination with cediranib, bevacizumab, and chemotherapy were approximately 17.0, 12.5 and 19.5 months, respectively.

**Conclusion:** PARPi combination therapy is more effective as maintenance treatment for ROC than monotherapy, and the efficacy of PARPis in combination with chemotherapy is higher than that of the combination with antiangiogenic drugs. We found that the PFS of BRCA wild-type was similar to that of HRD-positive patients, and there was no significant difference in PFS between olaparib, niraparib, and rucaparib, which provides necessary quantitative information for the clinical practice of PARPis in the treatment of ROC.

## Introduction

Ovarian cancer is a malignant gynecological tumor with a high mortality rate. It is traditionally treated with cytoreductive surgery and chemotherapy, but the recurrence rate in patients is high (Ma et al., 2020). Maintenance therapy for ovarian cancer fills the treatment gap between chemotherapy and disease progression, prolongs the time from chemotherapy to disease progression (Disilvestro and Alvarez Secord, 2018), and improves prognosis.

Poly (ADP-ribose) polymerase (PARP) inhibitors (PARPis) have ushered in a new era for the treatment of ovarian cancer. Generally, PARPis kill tumor cells by blocking single-strand DNA break repair and through PARP “trapping” in tumor cells that lack the ability to repair double-strand DNA damage, such as breast-related cancer antigens (BRCA) mutations and/or homologous recombination deficiency (HRD) (Kotsopoulos et al., 2016). As of August 2020, the FDA had approved three PARPis for the maintenance treatment of recurrent ovarian cancer (ROC): olaparib (Food and Drug Administrat, 2020a), rucaparib (Food and Drug Administrat, 2020b), and niraparib (Food and Drug Administrat, 2020c). However, there are still several challenges associated with the use of PARPis in clinical practice, such as the sensitivity of biomarkers for drug efficacy (BRCA genes and HRD status) (Rose et al., 2020), and the type of therapy (monotherapy administered alone or combination therapy as PARPis combination with chemo- or target-therapies) (Chan et al., 2020). Unfortunately, no research has comprehensively analyzed these issues.

Model-based meta-analysis (MBMA) is an effective method that can quantitatively compare the efficacy characteristics of drugs and deduce the impact of heterogeneity between trials on the results by establishing pharmacodynamic and covariate models (Mould, 2012). Compared to conventional meta-analysis, MBMA can utilize data more thoroughly and provide more abundant information (Upreti and Venkatakrishnan, 2019). In this study, MBMA was used to compare the efficacy features of PARPis and screen for reasonable covariates in order to quantitatively analyze the aforementioned issues, thereafter instructing PARPis’ clinical use.

## MethodS

### Search Strategy

We systematically searched PubMed, EMBASE, and the Cochrane Library from inception to February 6, 2020, for randomized clinical trials (RCTs). For the database search, we used “ovar* and cancer*, carcinoma*, neoplasm, or tumour and Olaparib, LYNPARZA, rucaparib, RUBRACA, niraparib, ZEJULA, AZD2281, AZD-2281, KU-0059436, CO-338, AG14699, MK4827” as search terms in all fields. The search was restricted to articles published in English.

### Inclusion and Exclusion Criteria

The inclusion criteria for the analysis were as follows: 1) studies on patients with ROC for maintenance treatment; 2) studies on drugs, including olaparib, rucaparib, and niraparib, approved by the FDA; 3) randomized studies and published progression-free survival (PFS) results of the PARPi arm; and 4) information on biomarkers (BRCA gene or HRD status).

The exclusion criteria were: 1) reviews, case reports, or meta-analyses; 2) conference abstracts; and 3) studies on non-maintenance therapy.

### Data Extraction

The following information was extracted using Microsoft Excel software (version 2016): 1) characteristics of the literature (authors, year of publication, DOI/PMID, and clinical trial registration number); 2) trial design (randomized information and blind information); 3) characteristics of participants (disease information, biomarker status, Eastern Cooperative Oncology Group (ECOG) status, and sample size); 4) treatment information including PARPi and combination drugs (generic drug name, dosage, dose unit, strength, and administration information); 5) PFS results.

All data were extracted from the included studies by two independent researchers. Any dispute was resolved through discussion with a third independent researcher. Digitization software Get Data (version 2.26) was used to obtain graphical data. When the extraction errors of the graphics data were higher than 2%, data extraction was repeated, and the mean values were used as the final results.

### Risk of Bias Assessment

The quality of the literature was assessed based on the Cochrane risk-of-bias criteria by two independent investigators, and each item was graded as low, high, or unclear. In total, seven items were used to evaluate bias in each trial, including random sequence generation, allocation concealment, blinding of participants and personnel, blinding of outcome assessment, incomplete outcome data, selective reporting, and other biases. The term “other bias” included trials sponsored by drug companies or studies in which the baseline characteristics of the intervention groups were not similar.

The quality of the literature was graded independently by two researchers, and inconsistencies were resolved by a third independent researcher.

### Model Building

The primary endpoint was PFS, which was analyzed using the parametric survival function. Four basic hazard ratio models, including the exponential, Gompertz, Weibull, and log normal models (Eqs 1–4) (Holford, 2013; Ding et al., 2020), were evaluated. Once the hazard ratio model was defined, the PFS data were fitted using Eq. 5. The inter-study variability and residual error were also considered during the establishment of the basic model (Eqs 6–8).

$$h_{\left(t\right)}=\lambda_{0}$$ (1)

$$h_{\left(t\right)}=\lambda_{0}\times {\text{e}}^{\beta \times t}$$ (2)

$$h_{\left(t\right)}=\lambda_{0}\times {\text{e}}^{\beta \times \ln\left(t\right)}$$ (3)

$$h_{\left(t\right)}=\frac{{\left(\sigma t\sqrt{2\pi }\right)}^{-1}e^{\left(-1/2Z^{2}\right)}}{1-\varphi \left(z\right)},\;Z=\frac{\ln\left(t\right)-\mu }{\sigma }$$ (4)

$$\text{S}\left(\text{t}\right)=\text{exp}\left(-\overset{t}{\underset{0}{\int }}h\left(t\right)dt\right)$$ (5)

$$P_{i}=P_{pop}\times e^{\eta_{i}}$$ (6)

$$Obs_{j,i}=Pred_{j,i}\times \left(1+SE_{j,i}\times \varepsilon_{\left(1\right)j,i}\right)+SE_{j,i}\times \varepsilon_{\left(2\right)j,i}$$ (7)

$$SE_{j,i}=\sqrt{\frac{Obs_{j,i}\times \left(1-Obs_{j,i}\right)}{N_{i}}}$$ (8)

The hazard function h_(t)_ (Eqs 1–4) is the instantaneous hazard of dying at time t, and in Eqs 1–3 λ_0_ and β represent the hazard rate at time 0 and change in the regression coefficient of the hazard rate over time, respectively. In Eq. 4, μ and σ are the median and standard deviation of the distribution, respectively. In Eq. 6, P_i_ is the individual prediction of the model parameter, and P_pop_ is the population prediction of the corresponding model parameter. η_i_ represents the inter-study variability, which is assumed to be normally distributed, with a mean of 0 and a variance of ω^2^. In Eq. 7, Obs_j,i_ represents the observed PFS rate at time point j in study i. Pred_j_,_i_ is the corresponding individual prediction of the final model. ɛ_(1)j_,_i_ and ɛ_(2)j_,_i_ are the proportional residual and added residual errors, respectively, at the time point j of study i, assumed to be normally distributed with a mean of 0 and variance of σ_1_^2^ and σ_2_^2^. The residual variability was weighed by the standard error of the corresponding observational data, which can be acquired by Eq. 8, where N_i_ represents the sample size of study i.

We established a covariate model to screen for factors that potentially affect PFS, such as age, ECOG status, biomarker status, and study design, including blindness and combination drug information. The missing information in each arm was imputed using the median value of the entire study population. Covariates missing proportions of more than 30% were not tested.

Continuous covariates are described in Eq. 9, and categorical covariates are described in Eq. 10.

$${\text{P}}_{pop}=P_{typical}\times e^{\left(COV-COV_{median}\right)\times \theta_{COV}}$$ (9)

$${\text{P}}_{pop}=P_{typical}\times \left(1+\theta_{COV}\right)$$ (10)

In Eqs 9, 10, P_pop_ is the population prediction of the corresponding model parameter, P_typical_ is the typical value of the model parameter, COV is the reported value of the screened covariate, COV_median_ is the median value of the screened covariate, and θ_cov_ is the scaling factor.

Forward and backward elimination approaches were used to test the covariates. The covariates were added stepwise to the model if the objective function value (OFV) decreased by > 2.71 (corresponding to *p* < 0.1). After defining the full model, the significance of each covariate was tested individually by removing them, one at a time, from the full model. Any covariate that failed to decrease OFV by > 3.84 (corresponding to *p* < 0.05) was removed from the model.

### Model Evaluation

Goodness-of-fit (GOF) plots, visual predictive check (VPC), and bootstrap were used to identify the model’s performance. GOF plots included the following scatterplots: observation (OBS) *vs.* individual prediction (IPRED), OBS *vs.* population prediction (PRED), conditional weighted residual errors (CWRES) *vs.* PRED, and CWRES *vs.* time.

The VPC was used to check whether the model was able to reproduce the variability and main trends of the observed data. Typically, in this method, 1,000 datasets based on the final model parameters are modeled using Monte Carlo simulations. The observed data are then compared with the 2.5, 50, and 97.5^th^ percentiles of the simulated data to assess the predictive capacity of the final model.

Bootstrap, which was performed by 1,000 NONMEM repetitions of the final model, was used to assess the robustness of the model. The bootstrap median parameter values and 95% percentile bootstrap confidence intervals (CIs) were compared with the respective values estimated from the original data. The estimated parameters were considered stable and less affected by any individual study if the values were comparable.

### Model Simulation

A single-arm meta-analysis was performed to summarize the model parameters of each arm using random-effects models according to different situations. The point estimates of the model parameters and their 90% CI in different scenarios were obtained. Next, the parameter values were randomly selected from the parameter distribution of each scenario using the Monte Carlo simulation method. The process was repeated 10000 times at each time point, and the median value and 90% CI of PFS at the different time points and different scenarios were obtained.

The simulation scenarios included the following: 1) PFS of patients after monotherapy or combination therapy; 2) PFS of patients with different biomarkers; 3) PFS of patients after treatment with different PARPis; 4) PFS of patients in combination therapy with different combination drugs; 5) PFS of platinum-sensitive patients (those who had no progression or relapse within 6 months after chemotherapy) and partially platinum-resistant patients (those who progressed or relapsed within 12 months after chemotherapy).

### Software

The modeling process was completed using NONMEM7.3 (Level1.0, ICON Development Solutions, New York, United States). First-order conditional estimation was used to estimate the model parameters, and the bootstrap was executed using Perl-Sons-Nonmem (PsN4.9.0). Model simulation, graph drawing, and meta-analysis were performed using the R software (version 3.6.1, The R Foundation of Statistical Computing, Vienna, Austria). The assessment of the literature quality of the RCTs was completed using RevMan (Version 5.4, Nordic Cochrane Center, Copenhagen, Denmark).

## Results

### Characteristics of the Included Studies

A total of 922 articles were retrieved from the PubMed, EMBASE, and Cochrane Library databases, 206 of which were included in the full-text analysis. A total of eight articles (Kaye et al., 2012); (Ledermann et al., 2014); (Oza et al., 2015); (Mirza et al., 2016); (Coleman et al., 2017); (Pujade-Lauraine et al., 2017); (Mirza et al., 2019); (Liu et al., 2019) met the inclusion criteria (Supplementary Figure S1; Supplementary Material).

Of the eight included studies, there were 1,169 patients with ovarian cancer who received PARPis. The age range of the patients was 53.5-67.0 years (median, 58.1 years). There were 501 patients receiving olaparib treatment, 302 receiving niraparib treatment, and 366 receiving rucaparib. Among them, 669 patients tested positive for BRCA mutations (BRCAm) and a 100 patients tested positive for wild-type BRCA (BRCAwt). 400 patients were HRD-positive. A total of 1,077 patients received monotherapy, and 92 patients received combination therapy. The combined drugs included anti-angiogenic drugs: 21 patients and 43 patients for bevacizumab and cediranib, respectively, and 28 patients received a PARPi combined with a chemotherapy drug. There were 64 partially platinum-resistant ROC patients and 1,105 platinum-sensitive ROC patients. Demographic characteristics are shown in Table 1. Detailed information of each study is shown in Supplementary Table S1 (Supplementary Material).

**TABLE 1:** Demographic characteristics of included patients.

	BRCAm	BRCAwt	HRDp
Treatment Group	9	3	4
Sample size	669	100	400
Age( yr)	58.0(53.5∼61.0)	61.0(57.8∼63.0)	66.0(58.0∼67.0)
Dosage( mg)			
Olaparib	600(400∼800)	800(400∼800)	—
Rucaparib	1,200	−	1,200
Niraparib	300	−	300
Combination			
yes/no	43/626	21/79	28/372
ECOG score			
0(%)	70.0(50.0∼84.0)	70.0(68.9∼74.0)	70.0
1(%)	28.0(15.0∼40.6)	28.0(18.0∼30.0)	28.0
Drug treatment			
Olaparib	401	100	0
Rucaparib	130	0	164
Niraparib	138	0	234

The overall quality of the included studies was high, and there was no obvious risk of bias. However, it should be noted that the four included studies were open-label trial designs (Supplementary Figure S2; Supplementary Material).

### Model Establishment

The log-normal hazard function model was finally selected as the basic hazard function model as per the OFV minimization and precision of model parameter estimation.

When covariates were examined, we found that the type of therapy (monotherapy or combination therapy) had a significant impact on the model parameter μ . The final model is expressed in Eq. 11, and the model parameters are listed in Table 2.

$$\begin{array}{l} \mu ,pop=2.96\times \left(1+\theta_{Combo}\right)\\ \mathrm{where:}\\ \theta_{Combo}=\{\begin{array}{cc} -0.215\; & monotherapy\\ 0\; & combination\;therapy \end{array} \end{array}$$ 11

**TABLE 2:** List of final model parameters, bootstrap results and sensitivity analysis.

	Estimate (SE%)	Bootstrap median (95% CI)	Sensitivity analysis^a^ (SE%)
Parameters			
σ	0.999 (4.1)	0.997 (0.921∼1.083)	1.030 (6.2)
μ	2.96 (3.4)	2.96 (2.68∼3.19)	3.48 (24.3)
Combo on μ	−0.215 (22.3)	−0.213 (−0.302∼0.108)	−0.265 (65.3)
Variability parameters			
η(σ)	0.142 (15.0)	0.134 (0.070∼0.171)	0.151(19.1)
η(μ)	0.159 (11.5)	0.150 (0.104∼0.186)	0.132 (25.5)
ε(add)	1.241 (20.4)	1.200 (0.456∼1.694)	1.453 (18.5)
ε(pop)	0.429 (26.5)	0.432 (0.269∼0.715)	0.446 (28.9)

a Delete all the data from open-label studies for sensitivity analysis.

### Model Assessment

The GOF plots of the final model show that OBS and PRED, as well as OBS and IPRED, are evenly distributed on both sides of the diagonal, and that the regression trend line and standard line coincide. The CWRES of most points are evenly distributed around the 0 line within 6, and the fitting line of CWRES *vs*. time and PRED almost coincide with the 0 line, indicating that the model fits the measured data at different time points or at different observations, without obvious bias (Supplementary Figure S3, Supplementary material).

The success rate of the bootstrap resampling of the model was 96.3%. In addition, the model parameter distribution obtained by the bootstrap was close to the estimated model parameter value from the original dataset (Table 2), indicating that the model parameter estimation was relatively robust and less affected by individual studies. The VPC results showed that the 95% CI of model prediction covered most of the measured values of drug efficacy, indicating that the model had good predictive performance (Supplementary Figure S4; Supplementary Material).

Sensitivity analysis showed that after deleting all the data from open-label studies, the estimation of the model parameters was consistent with the original data set, suggesting that the model was more robust and less affected by open-label studies(Table 2).

### Model Simulation

**FIGURE 1:**
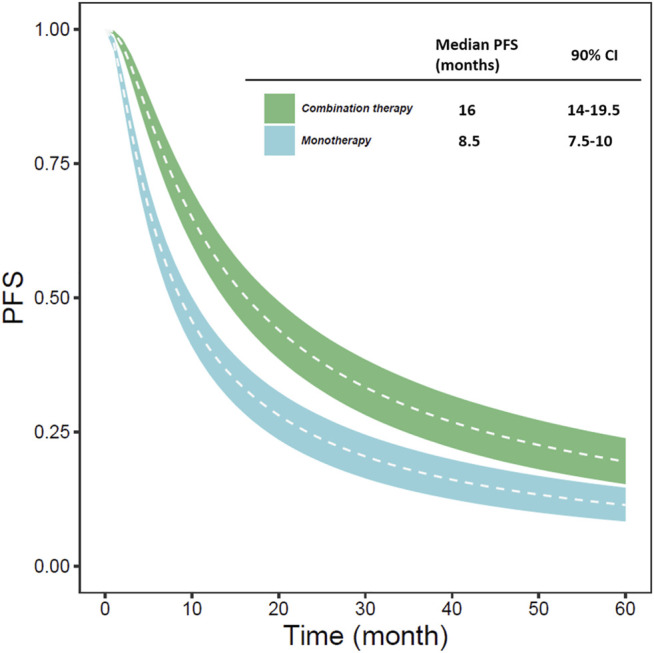
The typical predicted PFS of different types of treatment (monotherapy or combination therapy) (The white line represents the median PFS predicted by the model in each situation; the blue and green areas represent the 90% CI of the typical PFS predicted by the model).

Based on the final model, we estimated the typical PFS of different types of treatment (monotherapy or combination therapy) (Figure 1). Simulation results showed that the typical PFS of monotherapy and combination therapy was 8.5 months (90% CI: 7.5-10.0 months) and 16 months (90% CI: 14.0-19.5 months), respectively.

In this study, 501 subjects were treated with olaparib, 302 with niraparib, and 366 with rucaparib. The median PFS values of olaparib, niraparib, and rucaparib monotherapy were approximately 9.5, 10.5, and 12.0 months, respectively. For combination therapy, the median PFS values of olaparib, niraparib, and rucaparib were approximately 19.0, 20.0, and 25.0 months, respectively.

A total of 669 patients with BRCA mutations, 100 subjects with wild-type BRCA, and 400 HRD-positive subjects were included in the study. The median PFS of patients with BRCA mutation, wild-type BRCA subjects, and HRD-positive subjects were approximately 11.0, 7.5, and 9.0 months in monotherapy and 23.0, 14.0 and 17.5 months in combination therapy, respectively.

A total of 64 patients had partially platinum-resistant ROC and 1,105 patients had platinum-sensitive ROC. The median PFS of patients with platinum-sensitive ROC was approximately 11.0 and 22.0 months with monotherapy and combination therapy, respectively, and that of patients with partially platinum-resistant ROC was approximately 5.5 and 9.0 months with monotherapy and combination therapy, respectively.

**FIGURE 2:**
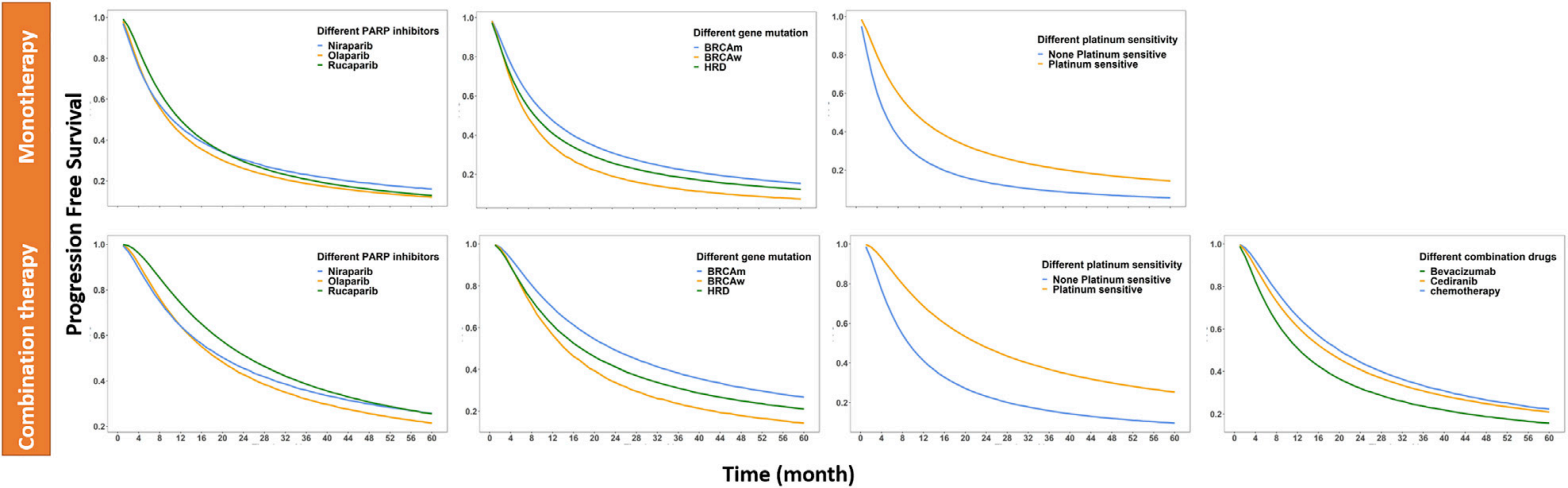
PFS of different simulated situations: corrected to monotherapy and corrected to combination therapy (The different simulation situations are different drug combinations, different PARP inhibitors, different biomarkers, and different platinum-sensitivity; the solid line represents the median of the drug effect predicted by the model in each situation).

In the combination study, patients treated with combination drugs, including antiangiogenic drugs such as bevacizumab and cediranib, were 28 and 44, respectively, and 20 were treated with chemotherapeutic drugs. The median PFS values in combination with cediranib, bevacizumab, and chemotherapy were approximately 17.0, 12.5 and 19.5 months, respectively. All the data are listed in Figure 2 and Table 3.

**TABLE 3:** Summary of different simulation results.

Simulation	Items	Median PFS/mons	95% CI of PFS/mons
Different PARP inhibitors			
Monotherapy	olaparib	9.5	7.0∼13.0
	niraparib	10.5	7.0∼17.0
	rucaparib	12.0	10.0∼14.0
Combination therapy	olaparib	19.0	13.0∼29.0
	niraparib	20.0	12.0∼37.0
	rucaparib	25.0	20.0∼31.0
Different biomarker status			
Monotherapy	BRCAm	11.0	9.0∼15.0
	BRCAwt	7.5	4.0∼18.0
	HRD-positive	9.0	7.0∼12.5
Combination therapy	BRCAm	23.0	17.5∼32.0
	BRCAwt	14.0	6.5∼42.5
	HRD-positive	17.5	12.0∼26.0
Different platinum-sensitivity			
Monotherapy	platinum-sensitive	11.0	9.5∼13.0
	partially platinum-resistant	5.5	4.5∼7.0
Combination therapy	platinum-sensitive	22.0	18.0∼28.0
	partially platinum-resistant	9.0	7.0∼12.0
Different combination drug	chemotherapy	19.5	14.0∼31.0
	cediranib	17.0	15.0∼21.0
	bevacizumab	12.5	10.0∼16.0

## Discussion

The use of PARPis for treating ovarian cancer brings new hope for doctors and patients. In this study, a pharmacodynamic model of PARPis was established for the first time to quantitatively compare and analyze the challenges of current clinical use, including monotherapy *vs.* combination therapy, different biomarker status (BRCA mutant *vs.* BRCA wild type *vs.* HRD positivity), and the effectiveness of the three approved PARPis. Compared to traditional analysis methods, the establishment of a pharmacodynamic model can be used to predict the survival time at any arbitrary time point, not limited to the median survival time and 1-year survival rate. Moreover, through the establishment of a covariate model, a variety of influencing factors can be quantitatively analyzed simultaneously, and the degree of influencing factors on the survival time can be assessed. This can provide quantitative information to implement PARPis clinically in the treatment of ROC.

Under the action of PARPis, ovarian cancer cells may develop drug resistance during progression; hence, they need to be used in combination with other drugs (Kubalanza and Konecny, 2020). To the best of our knowledge, this is the first study to use this type of modeling to compare the effectiveness of PARPis as monotherapy and combination therapy. Our study showed that the effectiveness of PARPis in combination therapy was better than that of monotherapy, demonstrating the significance and necessity of combination therapy. This conclusion is consistent with that of previous clinical trials. The NSGO-AVANOVA2/ENGOT-ov24 trial showed that niraparib plus bevacizumab significantly improved PFS compared with niraparib alone (Mirza et al., 2019), while another study showed that the combination of cediranib and olaparib significantly extended PFS compared with olaparib alone in relapsed platinum-sensitive ovarian cancer (Liu et al., 2019). Furthermore, the efficacy of PARPis in combination with different drugs was determined in this study, and the results showed that the efficacy of PARPis combined with chemotherapy drugs was better than that of combination therapy with bevacizumab and cediranib, and the efficacy of the combination therapy with bevacizumab was slightly lower than that of the other two drugs. Different studies have revealed that PARPis combined with chemotherapeutic drugs can produce a synergistic effect by interfering with DNA single-strand repair (Gupta et al., 2019; Min and Im, 2020), resulting in improved efficacy. Bevacizumab and cediranib are angiogenesis inhibitors, and preclinical studies have shown that anti-angiogenic drugs lead to hypoxia in tumors, downregulate the expression of homologous repair proteins, and inhibit homologous repair, thereby enhancing the sensitivity of PARPis (Liu et al., 2014). However, since the current reported studies of combination therapy are phase II studies with a limited sample size, the robustness of the above conclusions still needs to be confirmed by large clinical studies with large sample sizes.

Only about 15% of patients with advanced serous ovarian cancer have germline BRCA mutations, 6% have somatic BRCA mutations (Amin et al., 2020), and approximately 50% of patients have HRD-positive mutations (Ibrahim et al., 2020). The National Comprehensive Cancer Network (NCCN) recommends the detection of HRD as a biomarker of PARPis in patients with advanced serous ovarian cancer (Miller et al., 2020), indicating the effectiveness of clinically recognized PARPis in an HRD-positive population. This study showed that the median PFS of BRCA wild-type and HRD-positive patients in ROC monotherapy was similar, proving that the drugs were also effective for BRCA wild-type patients. This conclusion is consistent with Tomao’s meta-analysis (Tomao et al., 2019) on monotherapy for maintenance treatment in patients with platinum-sensitive ROC.

Stemmer et al. (Stemmer et al., 2020) qualitatively compared three approved PARPis (olaparib, rucaparib, and niraparib) for platinum-sensitive ovarian cancer patients. The results showed that the three approved PARPis had similar efficacy in terms of PFS. In this study, the median PFS values of olaparib, niraparib, and rucaparib monotherapy, as simulated by the model, were 9.5, 10.5, and 12.0 months, respectively. This difference may be due to the fact that niraparib is more selective to PARP1 and PARP2, while olaparib and rucaparib are selective to PARP1, PARP2, and PARP3, with a variety of activity (Thorsell et al., 2017). However, because PARPis interfere with different components of complex DNA repair networks in tumor cells, the difference in the effectiveness of the three PARPis decrease over time (Burdak-rothkamm and Rothkamm, 2020).

Patients with platinum resistance are generally considered to benefit less from further therapy[39]. Only 64 patients with partially platinum-resistant ROC were included for evaluation, despite platinum sensitivity not being a critical factor that could significantly affect efficacy. However, from the original data, it was obvious that efficacy in platinum-sensitive patients was better than that in non-platinum-sensitive patients (Supplementary Figure S5; Supplementary material). This study attempted to simulate the effectiveness of PARPis in patients who had progressed or relapsed within 12 months of chemotherapy, with a median PFS of 5.5 months in monotherapy. However, due to limited data (*n* = 64), clinical trials are necessary in the future for further confirmation.

Due to the limited information in the literature, there was no effectiveness data in HRD-negative patients; hence, this group of patients was not included as part of biomarker status in this study. To explore the effectiveness of PARP inhibitors in patients with platinum resistance, this group of patients was also included, but the sample size included in the model was limited (*n* = 64); therefore, the results need to be interpreted with caution. In addition, since the primary outcome of most trials on maintenance therapy in ROC is PFS, the available data on overall survival (OS) in clinical studies of PARPis are not comprehensive enough, and hence, only PFS data were analyzed in this study.

## Conclusion

A pharmacodynamic model of PARPis was established in this study to quantitatively evaluate the efficacy of PARPis as a maintenance treatment in ROC. This study found that combination therapy was more efficacious in maintenance therapy, and the efficacy of combined chemotherapy was better than that of combined antiangiogenic drugs. There was no significant difference in PFS between olaparib, niraparib, and rucaparib, and the PFS of wild-type BRCA was similar to that of HRD-positive patients. This study provides necessary quantitative information for the clinical use of PARPis in the treatment of ROC.

## Data Availability

The original contributions presented in the study are included in the article/Supplementary Material, further inquiries can be directed to the corresponding authors.
