# Supplementary material for: Quantitative Analysis of the Efficacy of PARP Inhibitors as Maintenance Therapy in Recurrent Ovarian Cancer
Source: Front Pharmacol. 2021 Nov 8;12:771836. doi: 10.3389/fphar.2021.771836 (PMC8606554; doi:10.3389/fphar.2021.771836)
Supplement: Supplementary file 1 [file DataSheet1.docx]

**Supplementary**


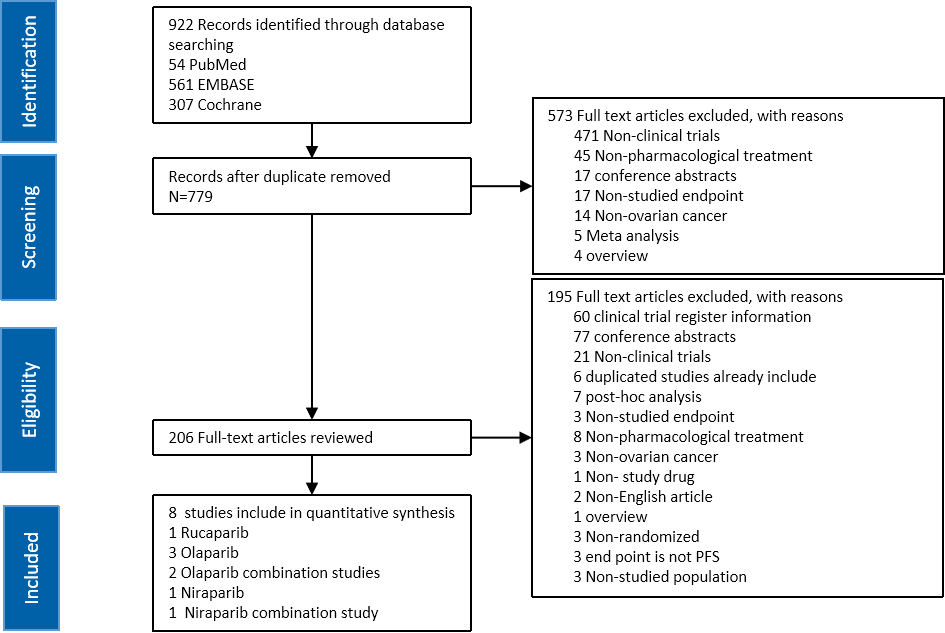


**Figure S1 Flowchart of literature search and the selection of the included studies**

**Table S1 Summary of the included studies***

| Reference | Treatment | Disease setting | Study type | Total N.of pts | N.of BRCAm/HRD positive pts | N.of BRCAw/HRD negative pts | ClinicalTrials.gov number |
| --- | --- | --- | --- | --- | --- | --- | --- |
| Ledermann2014 (study19)^13^ | Olaparib capsules,400mg BID | Platinum-sensitive recurrent OC | Phase II, double blind, RCT | 136 | 74 | 57 | NCT00753545 |
| Oza2014(Study41)^14^ | Olaparib capsules,200mgBID+chemo therapy group | Platinum-sensitive recurrent OC | Phase II, open-label, RCT | 81 | 20 | 3 | NCT01081951 |
| Pujade-Lauraine2017(SOLO2/ENGOT-Ov21)^15^ | Olaparib tablets,150mgBID | Platinum-sensitive recurrent OC | Phase III, double blind, RCT | 196 | 196(190germlineBRCA mutation) |  | NCT01874353 |
| Mirza2016(ENGOT-OV16/NOVA)^16^ | Niraparib tablets,300mgQD | Platinum-sensitive recurrent OC | Phase III, double blind, RCT | 372 | 138 | 234 | NCT01847274 |
| Kaye2011^17^ | Olaparib capsule,200mg/400mgBID | Recurred or progressed within 12months, of the most recent platinum-based chemo therapy regimen | Phase II, open-label, RCT | 32/32 | 32/32 |  | NCT00628251 |
| Coleman2017(ARIEL3)^18^ | Rucaparib tablets,600mgBID | Platinum-sensitive recurrent OC | Phase III, double blind, RCT | 375 | 130 | 245 | NCT01968213 |
| Liu2019^19^ | Olaparib capsules,400mgBID/Olaparib capsules,200mgBID+cediranib | Platinum-sensitive recurrent OC | Phase II, open-label, RCT | 46/44 | 24/23 | 22/21 | NCT01116648 |
| Mirza2019(NSGO-AVANOVA2/ENGOT-ov24)^20^ | Niraparib tablets 300mgQD+bevacizumab15mg/kg Q3W/Niraparib tablets 300mgQD | Platinum-sensitive recurrent OC | Phase II, open-label，RCT | 97 | 58pts with HRD postive | 39pts with negative or unknown HRD | NCT02354131 |

N.A. Not available; BID: twice daily

* the table only list the information of the arm receiving PARP inhibitor

(a)


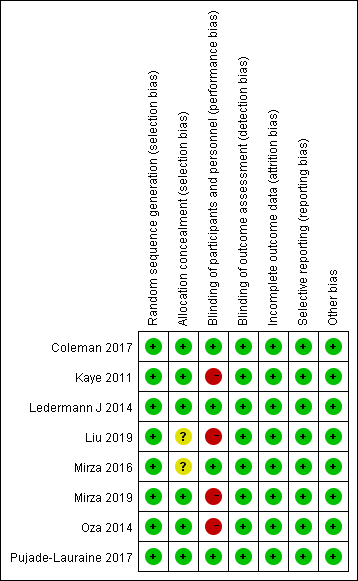


(b)


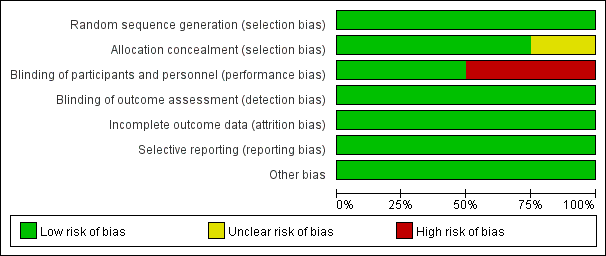


**Figure S2 Risk of bias assessment. Using Cochrane’s risk of bias assessment tool to assess the Study-level (a) and Overall (b) risk of bias.**


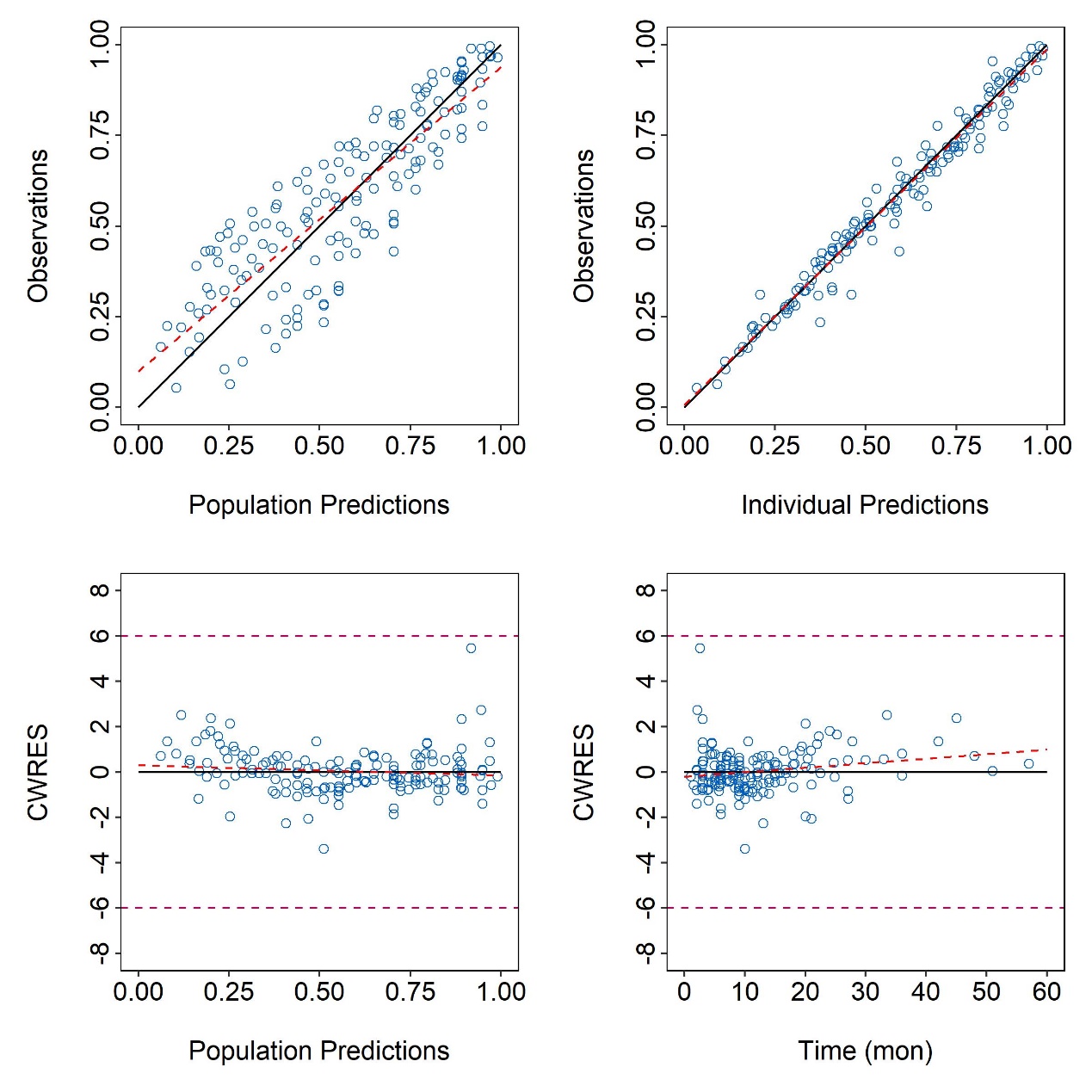


**Figure S3 Goodness-of-fit plot of the final model.**

(the upper two (from left to right) represent the observations vs PRED, the observations vs IPRED, the lower two scatter charts (from left to right) represent CWRES vs PRED, CWRES vs time. The red dotted line represents the fit line. Solid black lines in the upper two are diagonal, and pink dotted lines are 0 lines and ±6 reference lines in lower two, respectively.)


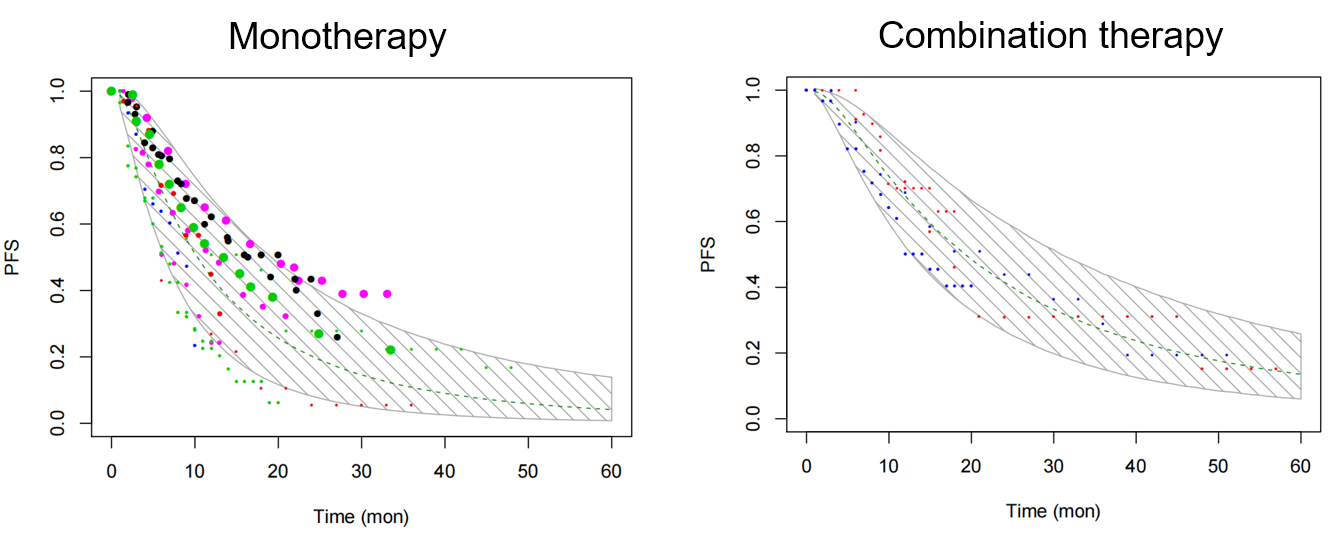


**Figure S4** VPC of the final model

(The shaded area represents the 95% CI of PFS predicted by the model, the green dotted line represents the median of the drug effect predicted by the model, the dot represents the measured value of the data, and the size of the dot is related to the sample size)


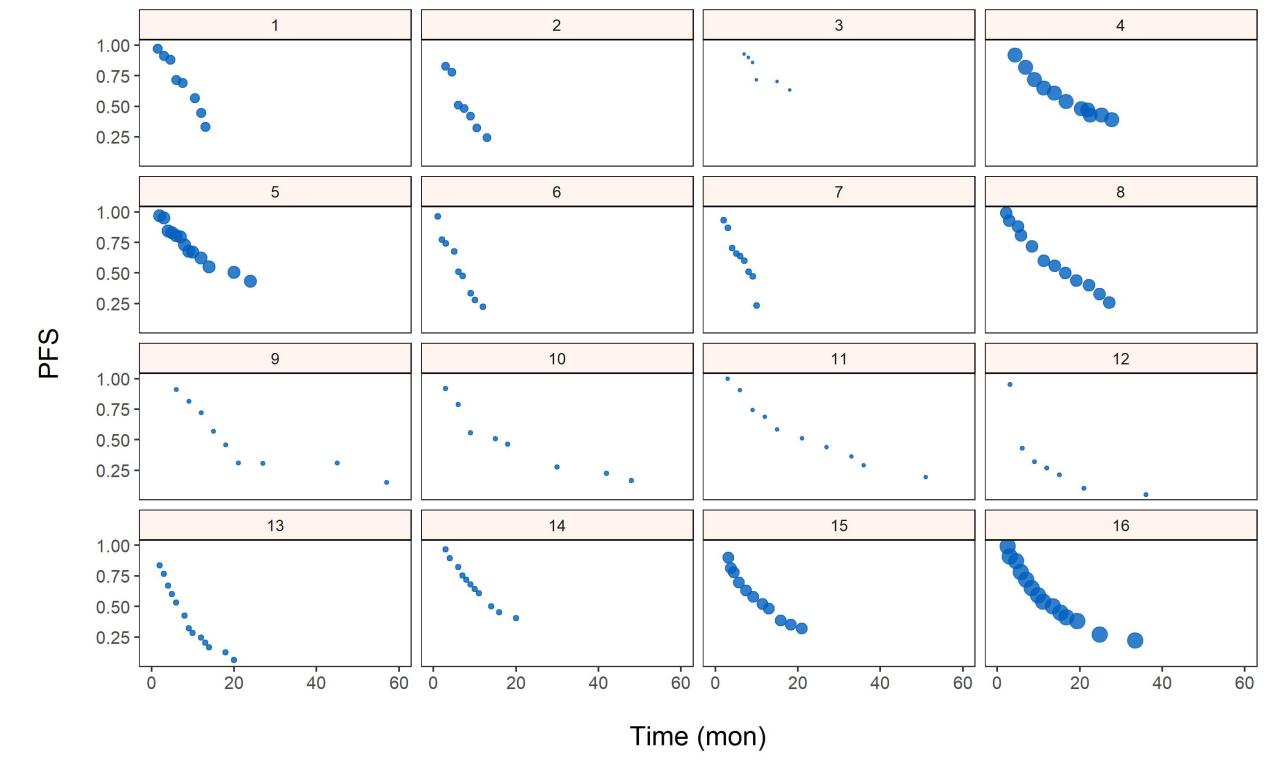


**Figur**e **S5** Original Data check

( ID 6 and 7 were data from partially platinum-resistant patients, all the others from platinum-sensitive patients)
